# Comparative Transcriptomic Profiling of Pellicle and Planktonic Cells from Carbapenem-Resistant *Acinetobacter baumannii*

**DOI:** 10.3390/antibiotics12071185

**Published:** 2023-07-13

**Authors:** Heng Kang Ng, Suat Moi Puah, Cindy Shuan Ju Teh, Nuryana Idris, Kek Heng Chua

**Affiliations:** 1 Department of Biomedical Science, Faculty of Medicine, Universiti Malaya, Kuala Lumpur 50603, Malaysia; elyoniselyon@gmail.com (H.K.N.); suatmoi@um.edu.my (S.M.P.); 2 Department of Medical Microbiology, Faculty of Medicine, Universiti Malaya, Kuala Lumpur 50603, Malaysia; cindysjteh@um.edu.my (C.S.J.T.); nuryana@um.edu.my (N.I.)

**Keywords:** carbapenem-resistant *Acinetobacter baumannii*, differential expressed genes, pellicle, planktonic, RNA-sequencing

## Abstract

*Acinetobacter baumannii* forms air–liquid interface pellicles that boost its ability to withstand desiccation and increase survival under antibiotic pressure. This study aims to delve into the transcriptomic profiles of pellicle cells from clinical strains of carbapenem-resistant *A. baumannii* (CRAB). The total RNA was extracted from pellicle cells from three pellicle-forming CRAB strains and planktonic cells from three non-pellicle-forming CRAB strains, subject to RNA sequencing using Illumina HiSeq 2500 system. A transcriptomic analysis between pellicle and planktonic cells, along with differential expression genes (DEGs) analysis and enrichment analysis of annotated COGs, GOs, and KEGGs, was performed. Our analysis identified 366 DEGs in pellicle cells: 162 upregulated genes and 204 downregulated genes. The upregulated ABUW_1624 (*yiaY*) gene and downregulated ABUW_1550 gene indicated potential involvement in fatty acid degradation during pellicle formation. Another upregulated ABUW_2820 (*metQ*) gene, encoding the D-methionine transporter system, hinted at its contribution to pellicle formation. The upregulation of two-component systems, CusSR and KdpDE, which implies the regulation of copper and potassium ions in a CRAB pellicle formation was also observed. These findings provide valuable insights into the regulation of gene expression during the formation of pellicles in CRAB, and these are potential targets that may aid in the eradication of CRAB infections.

## 1. Introduction

*Acinetobacter baumannii* is a clinically significant Gram-negative coccobacillus pathogen that poses a serious threat to immunocompromised patients in healthcare settings worldwide [1]. This pathogen accounts for a substantial proportion of hospital-acquired infections, causing severe diseases such as ventilator-associated pneumonia and bacteraemia [2]. The treatment of *A. baumannii* infections is challenging due to its propensity to acquire antimicrobial resistance determinants. This ability is due to the high genome plasticity of *A. baumannii*, which allows for the incorporation of exogenous DNA from the environment, primarily via horizontal gene transfer such as plasmid mediated conjugation and natural transformation [3]. Disturbingly, over 50% of carbapenem resistance in *A. baumannii* has been reported across Southeast Asian institutions [4]. In Malaysia, the National Surveillance of Antibiotic Resistance 2021 revealed alarming rates of *A. baumannii* resistance to carbapenems, with imipenem and meropenem resistance reaching 67.7% and 68.8%, respectively [5]. Recognising the urgency of the situation, the World Health Organisation has included carbapenem-resistant *A. baumannii* (CRAB) in their “Global priority list of antibiotic-resistant bacteria” in 2017 [6]. Adding to its clinical impact, *A. baumannii* demonstrates prolonged survival in hospital environments, attributed in part to its persistent tolerance to desiccation and increased survival under drug pressure. One key mechanism contributing to this trait is the formation of biofilms on various biotic and abiotic surfaces [7]. Bacterial biofilms are aggregations of bacterial cells attached onto a surface or to each other, forming complex structures and surrounded by a protective self-secreted matrix of extracellular polymeric substances [8,9]. While much attention is given to biofilm formation on solid surfaces, it is worth noting that *A. baumannii* is also capable of forming another type of biofilm at the air–liquid interface, known as pellicles [10,11,12]. Pellicles create a favourable niche for obligate aerobic *A. baumannii,* enabling direct access to abundant oxygen from the air and simultaneous nutrient acquisition from the surrounding media in a stagnant environment [12].

Several studies have provided evidence of the potential molecular mechanisms involved in the formation of pellicles by *A. baumannii*. For instance, the *csuA/BABCDE* pilus chaperone–usher assembly system responsible for pili production was identified in all clinical biofilm- and pellicle-forming *A. baumannii* strains using polymerase chain reaction screening assays [13]. The proteomic analyses conducted by Marti et al. [14] and Nait Chabane et al. [10] showed an alteration in the expression of the membrane proteins, including those involved in iron uptake systems, lipid and carbohydrate transport, cellular metabolism, starvation, porins, and pili, in *A. baumannii* pellicles compared to planktonic cells. Kentache et al. [15] reported the overexpression of proteins related to two-component systems (TCSs) and type VI secretion systems (T6SS) in 4-day-old pellicle *A. baumannii* cells, suggesting their involvement in regulating pellicle formation. Therefore, understanding the molecular mechanisms underlying *A. baumannii* pellicle formation is crucial for identifying potential therapeutic targets. In recent years, RNA-sequencing (RNA-seq) has become a powerful tool for transcriptomic analysis, enabling researchers to gain insights into the functional elements of the genome and the differential gene expression that drives cellular phenotypes [16]. Numerous studies have employed RNA-seq to identify the genes involved in *A. baumannii* biofilm and pellicle formation. Independent research groups from Canada, South Korea, and China have reported the regulatory roles of a response regulator (AvnR), a LysR-type transcriptional regulator (LeuO), and the *abaI/abaR* quorum sensing system in the biofilm and pellicle formation of *A. baumannii* [17,18,19].

Building upon our previous study, which identified the rare phenotypic trait of pellicle formation in clinical CRAB isolates collected from the intensive care units of the University Malaya Medical Centre. These isolates could be more virulent as they produced robust biomass in Mueller–Hinton broth with OD_570_ ranging from 1.689 ± 0.180 to 3.203 ± 0.569 [20]. Therefore, we aim to delve into the molecular mechanisms underlying the pathogenesis of these pellicle-forming isolates. More specifically, this study aims to investigate gene expression profiles through comparative transcriptomic analysis of pellicle and planktonic cells of CRAB using RNA-seq. By employing this approach, we seek to uncover novel insights into the genetic basis of pellicle formation, paving the way for potential therapeutic targets to combat CRAB infections.

## 2. Results

### 2.1. Differential Expression Genes Profiling

**Figure 1:**
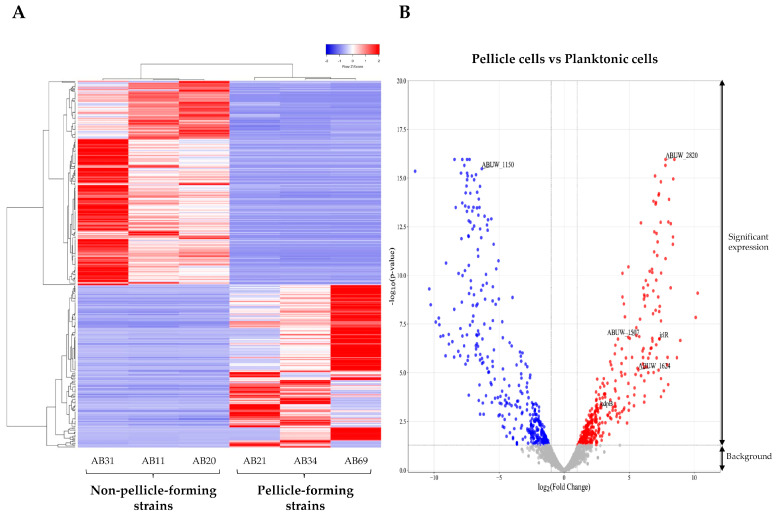
Differential expressed genes (DEGs) identified in the pellicle cells (AB21, AB34, and AB69) compared to planktonic cells (AB11, AB20, and AB31). (**A**) Expression heatmap of 366 DEGs with red representing a relatively higher level of expression and blue representing the relatively lower level of expression. (**B**) Volcano plot of DEGs with 162 upregulated genes (red dot) and 204 downregulated genes (blue dot). The grey dot signifies background genes with no significant expression changes.

A total of six samples (three pellicle cells from pellicle-forming CRAB strains and three planktonic cells from non-pellicle-forming CRAB strains) were subjected to RNA-seq. On average, 31,410,013 raw paired-reads with 4.712 Giga bases yield, Phred score Q30 of 83.58% and a length of 150 bps were obtained (Supplementary Table S1). After trimming the low-quality reads, adapter sequences, and bases below the quality threshold, an average of 31,392,514 reads were obtained with an average length of 116 bps. The established type strain *Acinetobacter baumannii* AB5075-UW (Gene accession no: CP008706.1) was used as the reference genome in this study as (i) it is a hypervirulent strain that was widely used in various studies [21], and (ii) it is a multidrug-resistant strain of clinical origin that was used for the evaluation of pathogenesis and antimicrobial treatments [22]. After the assembly of trimmed reads to the reference genome *Acinetobacter baumannii* AB5075-UW, an average of 14,930,220 paired reads were mapped. A total of 3,868 differential expression genes (DEGs) were detected after the CPM reads mapped analysis (Supplementary Table S2), while 366 DEGs (log_2_ fold change > 2 and <−2, *p*-value < 0.05, FDR *p*-value < 0.01), including 162 upregulated and 204 downregulated DEGs, were identified in the pellicle cells (Figure 1, Supplementary Table S3).

### 2.2. Cluster of Orthologous Groups Functional Classification of Proteins

**Figure 2:**
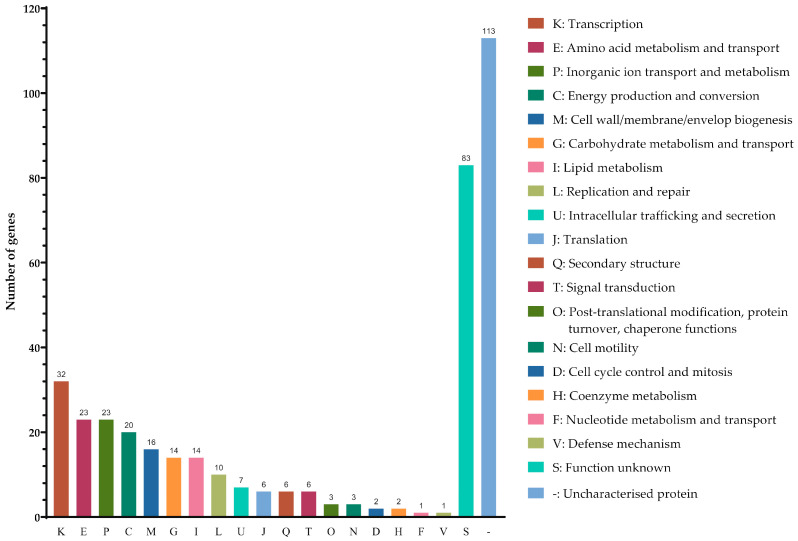
Classification of the differential expressed genes identified in pellicle cells of clinical carbapenems-resistant *Acinetobacter baumannii* strains into functional categories based on the Clusters of Orthologous Groups enrichment analysis.

Among the 366 DEGs examined, 8.7% (32/366) were classified into transcription (K), 6.3% (23/366) were clustered under amino acid metabolism and transport I and inorganic ion transport and metabolism (P), 5.5% (20/366) were identified as being associated with energy production and conversion (C), 4.4% (16/366) were linked to cell wall/membrane/envelope biogenesis (M), 3.8% (14/366) were recognised to be associated with carbohydrate metabolism and transport (G) and lipid metabolism (I), and 2.7% (10/366) were involved in replication and repair (L). In addition, 30.9% (113/366) of proteins remained uncharacterised, (−) and 22.7% (83/366) had unknown functions (S) (Figure 2, Supplementary Table S4).

### 2.3. Gene Ontology Enrichment Analysis

**Figure 3:**
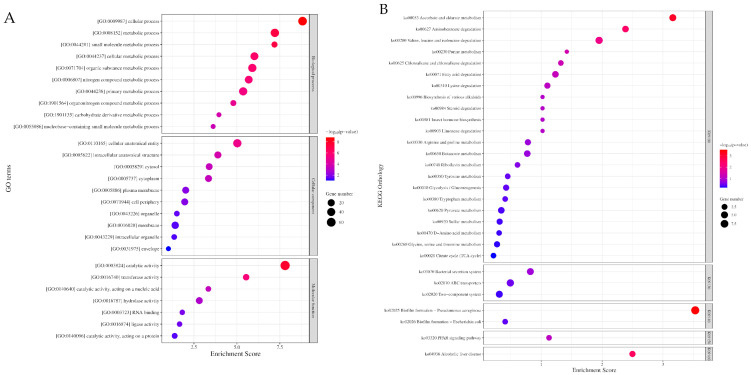
The enrichment of Gene Ontology (GO) and Kyoto Encyclopaedia Genes and Genomes (KEGG) of differential expressed genes identified in pellicle cells of clinical pellicle-forming carbapenem-resistant *Acinetobacter baumannii* strains. (**A**) Enriched GO terms of differential expressed genes (DEGs) in biological processes (Top 10), cellular components (Top 10), and molecular functions (Top 7) ontology group. (**B**) Top 30 enriched KEGG pathways of DEGs. (K09100: metabolism, K09130: environmental information processing, K09140: cellular processes, K01950: organismal systems, K01960: human diseases). The number of genes enriched in each GO term and KEGG pathway is shown as the size of the dot, and the adjusted *p*-value is shown as the colour gradient of the dot.

The Gene Ontology (GO) enrichment analysis yielded a total of 388 GO terms, which were further classified into three physiological function categories: biological processes (*n* = 264), molecular functions (*n* = 79), and cellular components (*n* = 45) (Supplementary Table S5a). In the biological process, the mainly enriched functional categories consisted of [GO:0009987] cellular processes and [GO:0008152] metabolic processes that further classified into [GO:0044281] small molecule (GO:0055086: nucleobase-containing), [GO:0044237] cellular, [GO:0071704] organic substance (GO:1901135: carbohydrate derivative), [GO:0006807] nitrogen compound (GO:1901564: organonitrogen) and [GO:0044238] primary metabolic processes. In cellular components, the mainly enriched category included the function of [GO:0110165] cellular anatomical entity that further classified into [GO:0005622] intracellular anatomical structure (GO: 0005829: cytosol; GO:0005737: cytoplasm), [GO:0016020] membrane (GO:0005886: plasma membrane; GO:0031975: envelope), [GO:0071944] cell periphery, and [GO:0016020] organelle (GO:0043229: intracellular organelle). In molecular functions, the mainly enriched functional category is [GO:0003824] catalytic activity (GO:0016740: transferase; GO:0140640: nucleic acid catalytic; GO:0140096: protein catalytic; GO:0016787: hydrolase; GO:0016874 ligase) and [GO:0003723] RNA binding (Figure 3A, Supplementary Table S5b).

### 2.4. Kyoto Encyclopaedia Genes and Genomes Enrichment Analysis

The Kyoto Encyclopaedia Genes and Genomes (KEGG) enrichment analysis revealed 55 KEGG Orthology (KO) terms (Supplementary Table S6a). Among these terms, the top 30 enriched KO terms can be further classified into five groups based on the BRITE hierarchy of KO terms (Figure 3B, Supplementary Table S6b): [K09100] metabolism, [K09130] environmental information processing, [K09140] cellular processes, [K09150] organismal systems, and [K09160] human diseases. Principally, the DEGs are involved in the metabolism pathway of amino acids; carbohydrates, lipids, and xenobiotics; ATP-binding cassette (ABC) transporters; secretion systems; two-component systems; and biofilm formation (Table 1).

#### 2.4.1. Metabolism Pathways

Among the amino acid metabolism pathways, the degradation of the branched-chain amino acids (valine, leucine, and isoleucine) pathway, designated as [ko00280], exhibited the highest number of DEGs, with a count of six. Within this pathway, five DEGs were found to be upregulated: ABUW_2506 (*hmgL1*), *hcaD*, ABUW_2453, ABUW_2456 (*hmgL2*), and ABUW_2455. Additionally, one DEG, namely, ABUW_1150, was downregulated. Furthermore, the metabolism pathways of other amino acids including lysine, arginine, proline, glycine, serine, threonine, tyrosine, and tryptophan were also found to be involved in the differential expressed gene set.

In additional to amino acid metabolism, the DEGs in this study were found to be involved in the pathway of metabolising carbohydrates, specifically, the pathways related to the metabolism of ascorbate, aldarate, pyruvate, butanoate, glycolysis/gluconeogenesis (glucose), and the citrate cycle. Within the [ko00053] ascorbate and aldarate metabolism pathway, four upregulated DEGs were identified: *gudD*, ABUW_2787 (*aldH*), *kdgD*, and *garD*. Additionally, one downregulated DEG, namely, ABUW_1150, was observed. Regarding the [ko00620] pyruvate metabolism pathway, three upregulated DEGs were detected: ABUW_1624 (*yiaY*), *ald1*, and ABUW_0255 (*aarC*), while one downregulated DEG: ABUW_1150 was identified. For the [ko00650] butanoate metabolism pathway, three upregulated DEGs: ABUW_0255 (*aarC*), ABUW_2506 (*hmgL1*), and ABUW_2456 (*hmgL2*), and one downregulated DEG: *gabD2,* were identified.

Furthermore, the DEGs in this study were found to be enriched in the [ko00071] fatty acid degradation pathway. Within this pathway, two DEGs were upregulated: *hcaD* and ABUW_1624 (*yiaY*), while two DEGs were downregulated: ABUW_1150 and ABUW_1151. The DEGs were also identified in the pathway of xenobiotics degradation. In the [ko00627] degradation pathway of aminobenzoate, three DEGs were upregulated: *hcaB*, *vanA,* and *vanB,* while one DEG: ABUW_1978 (*ethA*) was downregulated. For the [ko00625] chloroalkane and chloroalkene degradation pathway, ABUW_1624 (*yiaY*) was found upregulated, and ABUW_1150 was downregulated.

#### 2.4.2. Environmental Information Processing Pathways

Six enriched DEGs were involved in the ABC transporters with ABUW_2820 (*metQ*), the only one DEG was found upregulated; while the remaining five DEGs: ABUW_1570 (*ssuA*), ABUW_1569 (*ssuC*), *hisJ, hisM,* and *hisQ,* were downregulated. Additionally, five downregulated DEGs: ABUW_2618 (*vgrG*), ABUW_2578 (*hcp*), *icmF* (*impL/vasK*)*,* ABUW_2566 (*impK/ompA/vasF/dotU*), and *clpV* (*vasG*) were found to be involved in the type VI secretion system. In the two-component systems, three upregulated DEGs: ABUW_1507 (*cusS/copS/silS*), *irlR* (*cusR/copR/silR*), and *kdpB,* and two downregulated DEGs: *pfeA* (*fepA/iroN/pirA*) and *wza* (*gfcE*), were identified.

#### 2.4.3. Biofilm Formation Pathways

The analysis of DEGs revealed enrichment in the pathways related to biofilm formation, specifically based on ko02025: *Pseudomonas aeruginosa* and ko02026: *Escherichia coli*. Within these pathways, a total of 11 genes were found to be downregulated: ABUW_2572 (*impM*), *icmF* (*impL/vasK*), ABUW_2567 (*impJ*), ABUW_2568 (*impA*), ABUW_2580 (*impB*), ABUW_2579 (*impC*), ABUW_2578 (*hcp*), ABUW_2575 (*impH*), *clpV* (*vasG*), *wza* (*gfcE*), and ABUW_1145 (*gcvA*). Interestingly, 9 out of 11 genes were also associated with the type VI secretion system, while the remaining 2 genes: *wza* and *gcvA,* encode for a polysaccharide biosynthesis protein and a LysR family transcriptional regulator, respectively.

## 3. Discussion

**Figure 4:**
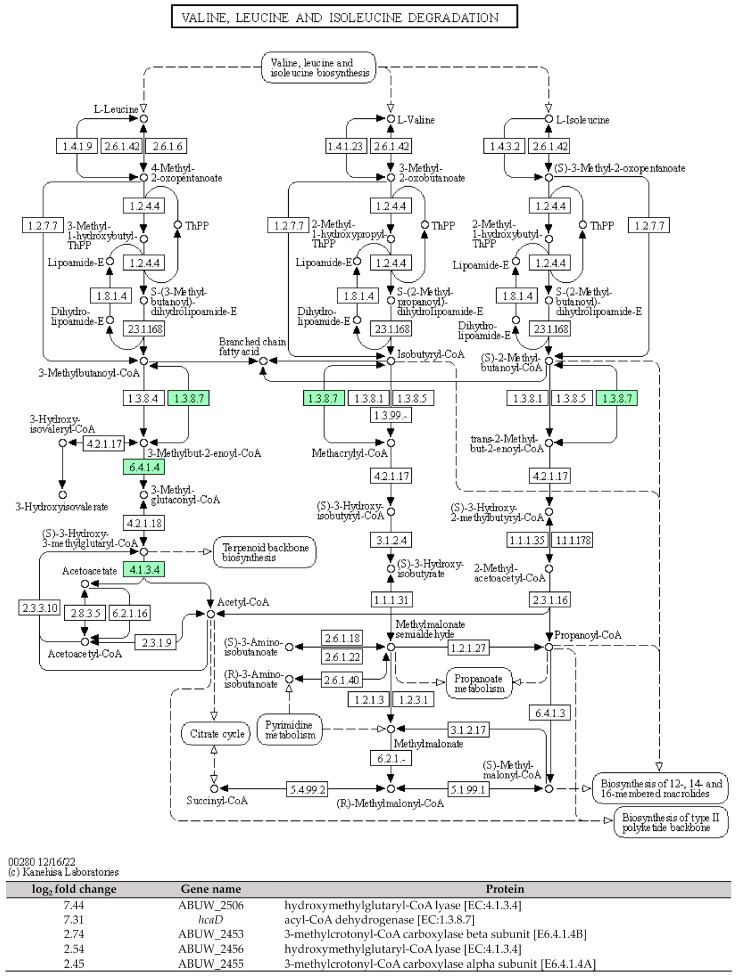
The KEGG pathway of the branched-chain amino acids (valine, leucine, and isoleucine) degradation and the involved differential expressed genes identified in pellicle cells of clinical carbapenem-resistant *Acinetobacter baumannii* strains. Green denotes the mentioned proteins. (The KEGG pathway map was obtained from the Kanehisa Laboratories.).

The capability of CRAB to form the air–liquid biofilms known as pellicles serves a dual purpose. First, it protects the bacteria from desiccation in clinical settings. Second, it provides a favourable niche for the direct acquisition of oxygen from the air while absorbing nutrients from the liquid media. Therefore, this unique adaptation can lead to clinical challenges, including drug resistance [23]. In this study, we identified five DEGs that encode enzymes involved in the degradation of branched-chain amino acids (valine, leucine, and isoleucine). These DEGs were found to be upregulated in the pellicle cells of CRAB strains, with log_2_ fold changes ranging from 2.45 to 7.44 (Figure 4). The enzymes identified include hydroxymethylglutaryl-CoA lyase [EC:4.1.3.4], acyl-CoA dehydrogenase [EC:1.3.8.7], 3-methylcrotonyl-CoA carboxylase alpha subunit [E6.4.1.4A], and 3-methylcrotonyl-CoA carboxylase beta subunit [E6.4.1.4B].

The research compiled over the years has consistently demonstrated the involvement of branched-chain amino acid metabolism in the remodelling of microbial biofilms [24,25,26]. For instance, a study conducted in China observed that *Pseudomonas aeruginosa* PAO1 biofilms at the 48-h mark exhibited the highest activity of *P. aeruginosa* aminopeptidase (*PaAP*), a leucine aminopeptidase responsible for leucine hydrolysis. Deletion of the *PaAP* gene using an in-frame deletion method resulted in bacterial cell death during the late stages of *P. aeruginosa* biofilm formation and led to disruptions in the biofilm structure [25]. More recently, a study conducted in the USA reported that the deletion of the *PaAP* gene in *P. aeruginosa* PAO1 led to a significantly increased cellular biomass (~10 µm^3^/µm^2^), but a lower concentration of the matrix exopolysaccharide (~0.2 Psl/biomass) and a less robust biofilm architecture compared to the wild type [26]. Therefore, it is speculated that aminopeptidase plays a role in regulating substantial changes in the matrix composition of pellicle biofilms in CRAB.

**Figure 5:**
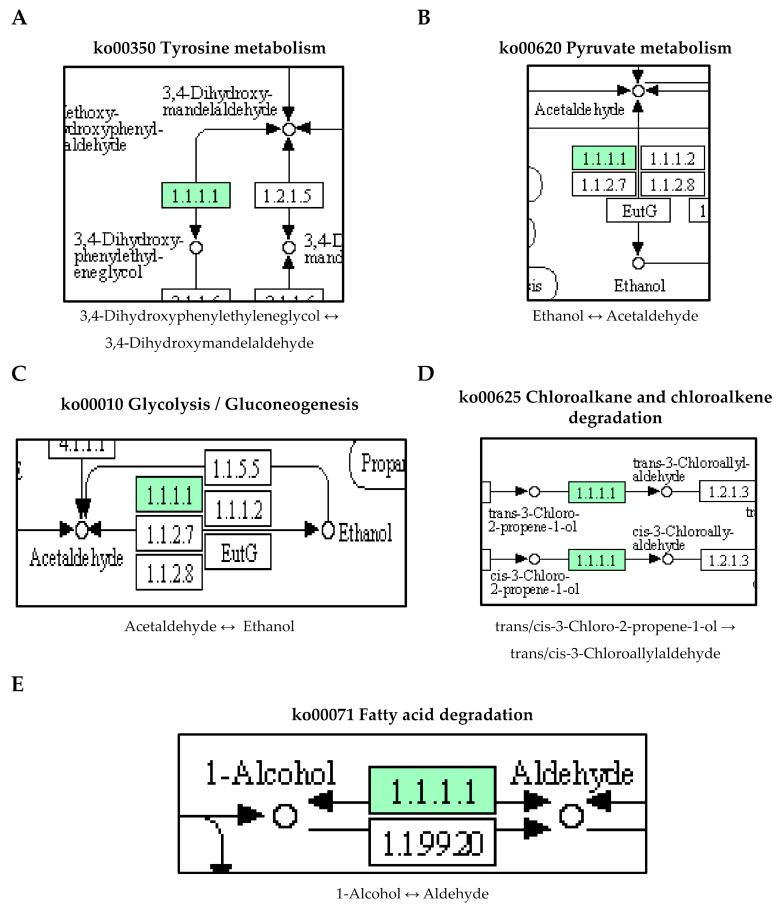
The upregulated ABUW_1624 (*yiaY*) gene detected in pellicle cells of clinical carbapenem-resistant *Acinetobacter baumannii* strains. Green denotes the mentioned proteins. (**A**) Tyrosine metabolism. (**B**) Pyruvate metabolism. (**C**) Glycolysis/Gluconeogenesis. (**D**) Chloroalkane and chloroalkene degradation. (**E**) Fatty acid degradation. (The partial KEGG pathway maps were obtained from the Kanehisa Laboratories.).

Additionally, our study revealed that the ABUW_1624 (*yiaY*) gene, which encodes for an alcohol dehydrogenase [EC:1.1.1.1] exhibited significant upregulation in pellicle cells compared to planktonic cells, with a log_2_ fold change of 5.71. ABUW_1624 (*yiaY*) is involved in multiple pathways, including tyrosine metabolism, pyruvate metabolism, glycolysis, chloroalkane and chloroalkene degradation, and fatty acid degradation (Figure 5). The upregulation of the *yiaY* gene was observed during the biofilm formation of pathogenic *Escherichia coli*, with a fold change of 7.21 [27]. Similarly, in *Acinetobacter johnsonii*, the upregulation of the *yiaY* gene associated with the tyrosine metabolism was linked to the activation of protein–tyrosine kinases and protein–tyrosine phosphatase in the stress response to tetracycline [28]. Therefore, these findings suggest that the expression of the *yiaY* gene in pellicle-forming CRAB not only potentially plays a regulatory role in pellicle biofilm formation but may also contribute to the stress response to antibiotics through the tyrosine metabolism pathway.

**Figure 6:**
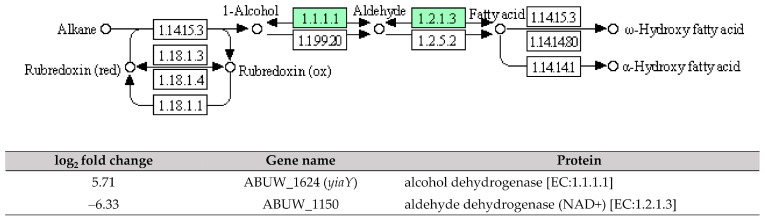
The interlinkage between upregulated ABUW_1624 (*yiaY*) and downregulated ABUW_1150 in the fatty acid degradation pathway. Green denotes the mentioned proteins. (The partial KEGG pathway map was obtained from the Kanehisa Laboratories.).

Furthermore, our study also identified an interlinkage between the upregulated ABUW_1624 (*yiaY*) gene and a downregulated ABUW_1150 gene (log_2_ fold change = −6.33) in pellicle cells. The ABUW_1150 gene encodes an aldehyde dehydrogenase (NAD+) [EC:1.2.1.3] involved in the fatty acid degradation pathway (Figure 6). This suggests a high conversion activity from 1-alcohol to aldehyde but a low conversion activity from aldehyde to fatty acid. The study reported the overexpression of fatty acid metabolism proteins (acetyl-CoA synthetase/AMP-fatty acid ligase, acyl-CoA dehydrogenase, and enoyl-CoA hydratase/isomerase) in 1-day-old pellicle cells of *A. baumannii* strain ATCC 17978, speculating its association with the biosynthesis of acyl-homoserine lactones in quorum sensing [15]. Furthermore, *cis*-2-decenoic acid, a short chain fatty acid, was identified as a signalling molecule capable of inducing the biofilm dispersion in P. aeruginosa PAO1, *E. coli*, *Klebsiella pneumoniae*, *Proteus mirabilis*, *Streptococcus pyogenes*, *B. subtilis*, *Staphylococcus aureus,* and *Candida albicans* [29,30]. Hence, it would be reasonable to conduct further investigations on the role of ABUW_1624 (*yiaY*) and ABUW_1150 in CRAB pellicle development using gene knockout methods.

**Figure 7:**
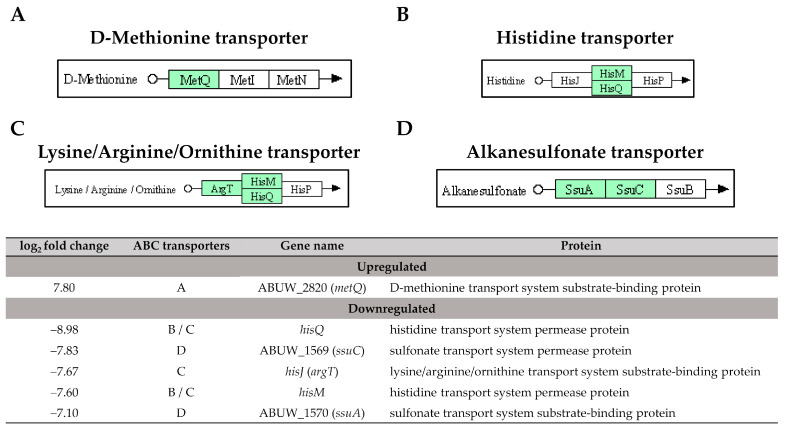
The identified ATP-binding cassette transporters in pellicle cells of clinical carbapenem-resistant *Acinetobacter baumannii* strains. Green denotes the mentioned proteins. (**A**) D-methionine transporter. (**B**) Histidine transporter. (**C**) Lysine/Arginine/Ornithine transporter. (**D**) Alkanesulfonate transporter. (The partial KEGG pathway maps were obtained from the Kanehisa Laboratories.).

ABC transporters play a crucial role in achieving cellular homeostasis by utilising ATP binding to export multiple substrates, including antibiotics during the bacterial stress response [31]. In this study, ABUW_2820 (*metQ*), which encodes the substrate-binding protein for the D-methionine transporter system, was observed being upregulated in pellicle cells with a log_2_ fold change of 7.80 (Figure 7). The previous research has demonstrated that a mixture of D-amino acids, including D-methionine and D-tyrosine, can inhibit the biofilm formation in *Staphylococcus aureus* and *Pseudomonas aeruginosa* by disrupting amyloid fibres that connect cells within the biofilm [32]. Another recent study on *Acinetobacter baumannii* ATCC 17978 shows that a transposon insertional mutant of *met*-related genes, specifically *metG*, significantly decreased pellicle biomass production [33]. Based on these findings, it is hypothesised that ABUW_2820 (*metQ*) may play a role in pellicle development, as indicated by the involvement of *met*-related genes. Further investigation is warranted to unravel its specific contribution to pellicle formation.

**Figure 8:**
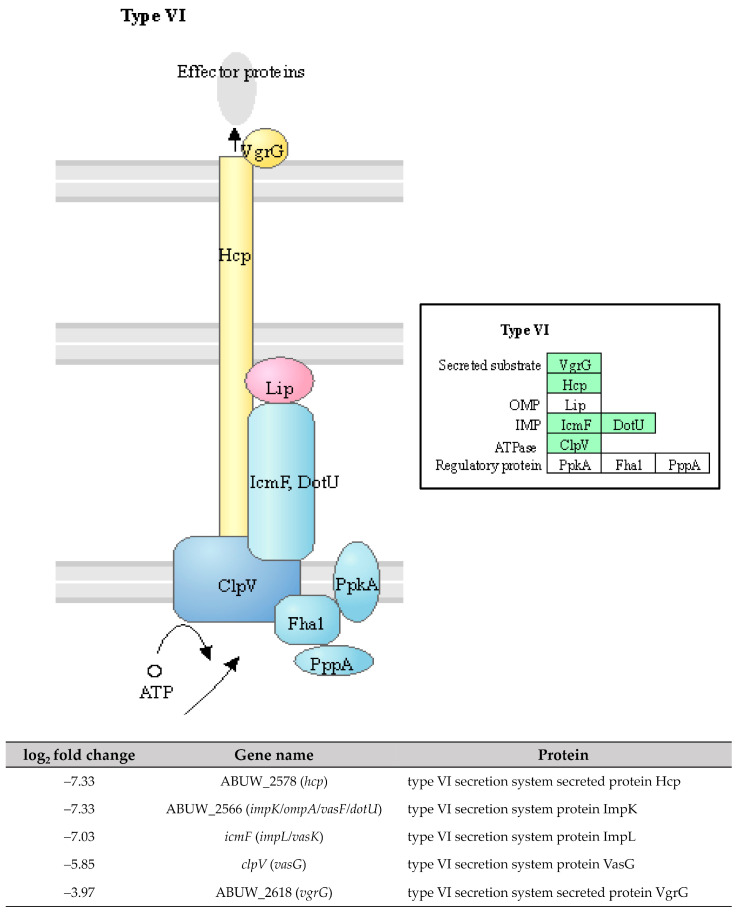
The five downregulated DEGs identified in pellicle cells of clinical carbapenem-resistant *Acinetobacter baumannii* strains involved in the type VI secretion system. Green denotes the mentioned proteins. (The partial KEGG pathway map was obtained from the Kanehisa Laboratories.).

The type VI secretion system (T6SS) is a phage-related system responsible for the secretion of toxic effector molecules that can kill both prokaryotic and eukaryotic cells, leading to cell death [34]. In our study, the identified DEGs involved in the T6SS were all downregulated with a log_2_ fold change ranging from −3.97 to −7.33 (Figure 8). This suggests a lower expression of T6SS in the pellicle cells compared to planktonic cells. The role of T6SS for biofilm forming ability in *A. baumannii* remains a subject of debate. A study conducted by Kim et al. [35] from Korea reported that T6SS positive *A. baumannii* isolates produced more biofilm mass compared to T6SS negative isolates, suggesting a positive correlation between T6SS and biofilm formation. However, contradictory findings were reported by Dong et al. [36] from China. The authors generated a deletion mutant of the *hcp* gene that caused the loss of T6SS function, thus, a significant increase in biofilm formation was observed in A. baumannii mutant compared to the wild type. Another two studies reported that the deletion of tssM, a type VI secretion system membrane subunit in *A. baumannii* DSM30011 and ATCC 17978 strains did not impact biofilm formation compared to their respective wild types [37,38]. Overall, the results of T6SS on biofilm formation in *A. baumannii* are inconsistent.

**Figure 9:**
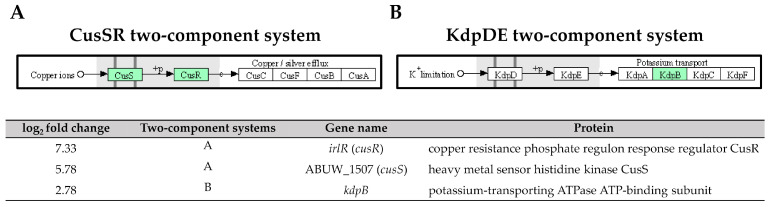
Three upregulated genes identified in pellicle cells of clinical carbapenem-resistant *Acinetobacter baumannii* strains involved in the (**A**) CusSR and (**B**) KdpDE two-component systems. Green denotes the mentioned proteins. (The partial KEGG pathway map obtained from the Kanehisa Laboratories.).

The two-component systems (TCS) are essential signal transduction pathways that enable bacteria to sense and respond to environmental cues through the coordinated action of histidine kinases and response regulators [39]. In our study, high expressions of ABUW_1507 (*cusS*) and *irlR* (*cusR*) genes (log2 fold change = 5.78 and 7.33) encoding the CusS sensor histidine kinase and the CusR response regulator, respectively, were observed in the pellicle-forming CRAB group (Figure 9). These genes are part of the CusSR TCS, which is involved in copper homeostasis regulation. Copper is an essential trace element required for numerous enzymatic reactions involved in bacterial electron transfer processes. However, high copper concentrations can result in cellular damage by interacting with lipids, proteins, nucleic acids, and other macromolecules [40]. Interestingly, transposon insertion mutants of *cusS* and *cusR* in *A. baumannii* AB5075 exhibited attenuated virulence in both *Galleria mellonella* larvae and murine models, highlighting the importance of this TCS in the pathogenicity of *A. baumannii* [41].

Another TCS, the *kdpB* gene, encodes for the potassium-transporting ATPase ATP-binding subunit in the KdpDE TCS, was also upregulated with a log_2_ fold change of 2.78 in pellicle cells of the CRAB strains (Figure 9). The KdpDE TCS consists of the membrane-bound histidine kinase sensor KdpD, which autophosphorylates in response to low potassium conditions. It then transfers the phosphoryl group to the cytoplasmic response regulator KdpE, which, in turn, promotes the transcription of K+ uptake systems, ensuring intracellular K+ homeostasis [42]. The studies involving the deletion of K+ transporters, including the KdpDE TCS, in *A. baumannii* ATCC 19606 have demonstrated a significant attenuation in virulence. The survival rate of *G. mellonella* larvae infected with the deletion mutant was only 20% within the first 24 h, compared to 60% for the wild-type strain [43]. Furthermore, the recent research has demonstrated that inhibiting K+ channels hampers biofilm formation in *A. baumannii* RS307, suggesting a correlation between membrane potential changes and K+ channels during biofilm development [44]. These findings consistently highlight the essential role of potassium ions and their respective channels in regulating virulence phenotypes, including biofilm formation.

Our study successfully elucidated the transcriptomic profiles from the pellicle cells of three pellicle-forming CRAB strains compared to the planktonic cells of three non-pellicle-forming CRAB strains. Although the small sample size is a limitation for this study, the knowledge of the underlying mechanisms driving pellicle formation, a rare trait in CRAB, holds significant importance in the field of antibiotic resistance research. The further assessment of their functional role in pellicle formation, as well as carbapenem resistance, requires future experimental validation.

## 4. Materials and Methods

### 4.1. Bacterial Strains and Culture Conditions

The clinical CRAB strains used in this study, including 3 pellicle-forming strains: AB21 (bronchoalveolar lavage), AB34 (sputum), and AB69 (tracheal secretion), and 3 non-pellicle-forming strains: AB11, AB20, and AB31, were recovered from sputum. The pellicle-forming ability of these strains was confirmed through a pellicle formation assay and impedance-based xCELLigence real-time cell analysis using an Agilent xCELLigence system (Agilent, Santa Clara, CA, USA) [20].

### 4.2. Pellicle and Planktonic Growth Conditions

All 6 CRAB strains were revived on Luria-Bertani agar plates (BD Difco, Grayson, GA, USA), and single colonies were inoculated into Mueller–Hinton (MH) broth (BD Difco, Grayson, GA, USA). The cultures were incubated overnight at 37 °C with an agitation speed of 150 rpm. The overnight cultures were diluted 1:100 into MH broth and incubated at 37 °C with an agitation speed of 150 rpm and were allowed to grow for 3.5 h until reaching the log phase, characterised by an OD600 of 0.6. Once the desired log-phase density was achieved, the cultures were further diluted to an OD_600_ of 0.1 using MH broth in borosilicate glass beakers. Each beaker contained a volume of 200 mL, providing sufficient space for biofilm formation. The cultures were then incubated statically at 37 °C for 48 h in the absence of light.

### 4.3. Cell Harvesting

The pellicles produced by pellicle-forming strains were formed on top of the culture and far from the bottom of the beaker, thus, pellicle cells were collected from the surfaces of the culture. The planktonic cells from non-pellicle-forming strains were collected from the 1/3 upper phase of bacterial culture. The collected cells were subjected to centrifugation with a centrifugation speed of 2600× *g* for 10 min at 4 °C. The harvested pellets were washed twice with 5 mL of chilled sterile phosphate-buffered saline solution, and the pellets were treated with RNAprotect bacteria reagent (Qiagen, Hilden, Germany) according to the manufacturer’s instructions. The treated cells were stored at −80 °C for further experiments.

### 4.4. RNA Extraction and Quality Assessment

For RNA extraction, 0.1 g of the previously treated cells of each strain were resuspended in TE buffer (30 mM Tris·Cl-1 mM EDTA, pH 8.0) containing 15 mg/mL lysozyme and 5% (*v*/*v*) of proteinase K solution and incubated for 10 min at room temperature for cell lysis. The total RNA was extracted and purified using RNeasy Mini kit (Qiagen, Hilden, Germany) and further treated with RNase-Free DNases Set (Qiagen, Hilden, Germany) for DNA removal. The purified total RNA was stored at −80 °C for further experiments. The quality of the purified total RNA was checked using bleached agarose gel electrophoresis [45]. A 1% (*w*/*v*) agarose gel was prepared with the addition of 1% (*v*/*v*) Clorox bleach in 1X Tris-acetate-EDTA (TAE) buffer. The purity of the RNA (OD_260/280_) was measured using a NanoDrop 2000 UV-Vis spectrophotometer (ThermoFisher Scientific, Denver, CO, USA). The concentration of the RNA was measured using a Qubit RNA Broad Range assay kit (Invitrogen, Waltham, MA, USA) for accurate quantification, and the RNA integrity was assessed by the RNA ScreenTape TapeStation system (Agilent, Santa Clara, CA, USA).

### 4.5. RNA-Sequencing Library Preparation and Illumina Sequencing

The cDNA libraries for RNA-sequencing of each strain were prepared using the hybrid of QIAseq Fast Select Bacterial—5S/16S/23S kit (Qiagen, Hilden, Germany) and QIAseq Stranded RNA Library Kit (Qiagen, Hilden, Germany). Briefly, Fast Select FH buffer was added to the purified total RNA for the removal of 5S, 16S, and 23S rRNA. The fragmentation of mRNA was then performed by incubating the mixture at multiple different temperatures and times following the manufacturer’s protocol. Next, the fragmentised mRNA was used as a template for the synthesis of first-strand cDNA, followed by the second-strand synthesis, and end-repaired, poly-A-tailing, and ligated with the unique combinatorial dual-index Y-adapters. Finally, CleanStart library amplification was performed and purified with QIAseq beads (Qiagen, Hilden, Germany) to obtain the final cDNA libraries.

The concentration and integrity of the final cDNA libraries were assessed using a Qubit dsDNA High Sensitivity assay kit (Invitrogen, Waltham, MA, USA) and D5000 ScreenTape Assay TapeStation system (Agilent, Santa Clara, CA, USA). Then, the accurate quantification of the final cDNA libraries was performed with real-time qPCR using QIAseq Library Quant assay kit (Qiagen, Hilden, Germany) with the ABI 7500 Fast real-time PCR system (ThermoFisher, Denver, CO, USA). Finally, the final cDNA libraries were sequenced with 150 bp paired-end on a HiSeq 2500 sequencing platform (Illumina, San Diego, CA, USA).

### 4.6. Bioinformatic Analysis of RNA-Seq Data

The raw reads obtained were cleaned by removing adaptor sequences, empty reads, and low-quality sequences and then normalised using CLC genomics workbench v12.0.3 (Qiagen, Hilden, Germany). The high-quality cleaned reads were aligned to the reference genome of *Acinetobacter baumannii* AB5075-UW (Gene accession no: CP008706.1) with parameters as follows: mismatch cost 2; insertion cost 3; deletion cost 3; length fraction 0.8, and similarity fraction 0.8. After alignment, the total gene counts were normalised using the weight trimmed mean of M values (TMM) method, and the expression values were converted into counts per million (CPM).

#### 4.6.1. Gene Differential Expression and Enrichment Analysis for RNA-Seq Data

The differential expression for the RNA-seq tool in the CLC genomics workbench v12.0.3 (Qiagen, Hilden, Germany) was used for identifying DEGs in pellicle cells compared to planktonic cells. Genes that were differentially expressed with *p*-value < 0.05 and FDR *p*-value < 0.01 were statistically significant, while the downregulated and upregulated DEGs were applied with the cut-off factor of a log_2_ fold change of <−2 and >2, respectively. The heatmap was generated using the hierarchy clustering method of average linkage with the distance measurement method of Spearman rank correlation via HeatMapper [46], whereas a volcano plot of DEGs was constructed using SRplot (www.bioinformatics.com.cn/en, accessed on 8 May 2023). An enrichment analysis was performed to identify orthologous genes and classify them into functional categories based on the similar ortholog alignment of their amino acid sequences (*E*-value < 0.001, Bit score > 60) based on the latest data from the clusters of orthologous groups (COG) database [47] using eggnog-MAPPER [48]. The COG bar chart was plotted using GraphPad Prism 8.0.2.

#### 4.6.2. Gene Ontology and Kyoto Encyclopaedia of Genes and Genomes Pathway Enrichment Analysis

The Gene Ontology (GO) enrichment analysis was performed based on Fisher exact test (*p*-value < 0.05) using Blast2GO 6.0.3. The GO terms of the DEGs were annotated using the InterProScan and GO-slim tools based on the latest EMBL-EBI database. For the Kyoto Encyclopaedia of Genes and Genomes (KEGG) enrichment analysis, the DEGs were annotated with the KEGG Orthology (KO) terms and pathways using bi-directional blast hit (Blast hit score > 60) on all available *Acinetobacter baumannii* genes set lists (Organism lists: *acb, abm, aby, abc, abn, abb*) in the KEGG Automatic Annotation Server (KAAS) [49]. Then, the KEGG enrichment analysis was performed based on the functional hypergeometric test [50] using the hypergeometric *p*-value test calculator provided by the Graeber Lab (https://systems.crump.ucla.edu/hypergeometric/, accessed on 8 May 2023). The GO and KEGG enrichment bubble graphs were plotted using SRplot (www.bioinformatics.com.cn/en, accessed on 8 May 2023).

## 5. Conclusions

In this study, a comparative transcriptomic analysis between pellicle cells and planktonic cells of clinical CRAB strains was performed, resulting in the identification of 366 DEGs with 162 genes upregulated and 204 genes downregulated in pellicle cells. Our findings provide valuable insights into several potential pathways associated with pellicle formation in CRAB. The upregulation of genes involved in the metabolism of branched-chain amino acids suggests their role in remodelling and promoting the formation of pellicle biofilms in CRAB. Additionally, the upregulation of ABUW_1624 (*yiaY*) and downregulation of ABUW_1150, both involved in fatty acid degradation may impact the synthesis of lipid membrane components in CRAB cells, subsequently influencing biofilm dispersal and the production of quorum sensing molecules. Furthermore, the high expression of the gene ABUW_2820 (*metQ*), which is involved in the D-methionine transporter system, as well as two-component systems: CusSR and KdpDE, suggests their potential involvement in the pellicle formation of CRAB. In summary, understanding the molecular mechanisms involved in pellicle development is crucial for the development of effective strategies to combat CRAB infections.

## Figures and Tables

**Table 1:** Top 30 enriched KEGG pathway of differential expression genes in the pellicle cells of clinical pellicle-forming carbapenem-resistant *Acinetobacter baumannii* strains.

KEGG Orthology	KEGG Pathway	Enrichment Score	*P*-Value	DEGs Number	Background Number	List of DEG	
						Upregulated	Downregulated
**K09100 Metabolism**							
K09105 Amino acid metabolism	ko00280 Valine, leucine, and isoleucine degradation	1.9510	0.0112	6	21	ABUW_2506, *hcaD*, ABUW_2453, ABUW_2456, ABUW_2455	ABUW_1150
	ko00310 Lysine degradation	1.1016	0.0791	3	11	ABUW_1519	*gabD2*, ABUW_1150
	ko00330 Arginine and proline metabolism	0.7820	0.1652	3	15	*astA2,* ABUW_2807	ABUW_1150
	ko00260 Glycine, serine, and threonine metabolism	0.2754	0.5303	3	29	*ydcW*, ABUW_1519	ABUW_1149
	ko00350 Tyrosine metabolism	0.4501	0.3547	2	13	ABUW_1624	*gabD2*
	ko00380 Tryptophan metabolism	0.4087	0.3902	2	14	-	ABUW_1150, ABUW_1948
K09101 Carbohydrate metabolism	ko00053 Ascorbate and aldarate metabolism	3.1606	0.0007	5	9	*gudD*, ABUW_2787, *kdgD*, *garD*	ABUW_1150
	ko00620 Pyruvate metabolism	0.3450	0.4518	4	36	ABUW_1624, *ald1*, ABUW_0255	ABUW_1150
	ko00650 Butanoate metabolism	0.7713	0.1693	4	23	ABUW_0255, ABUW_2506, ABUW_2456	*gabD2*
	ko00010 Glycolysis / Gluconeogenesis	0.4244	0.3764	3	23	ABUW_1624, *ald1*	ABUW_1150
	ko00020 Citrate cycle (TCA cycle)	0.2165	0.6075	2	21	ABUW_0255	*uca*
K09111 Xenobiotics biodegradation and metabolism	ko00627 Aminobenzoate degradation	2.3840	0.0041	4	8	*hcaB*, *vanA*, *vanB*	ABUW_1978
	ko00625 Chloroalkane and chloroalkene degradation	1.3229	0.0475	2	4	ABUW_1624	ABUW_1150
	ko00984 Steroid degradation	1.0218	0.0951	1	1	ABUW_2770	-
K09109 Metabolism of terpenoids and polyketides	ko00981 Insect hormone biosynthesis	1.0218	0.0951	1	1	-	ABUW_1150
	ko00903 Limonene degradation	1.0218	0.0951	1	1	-	ABUW_1150
K09102 Energy metabolism	ko00920 Sulfur metabolism	0.3177	0.4811	3	27	*msuE*	ABUW_1570, ABUW_1569
K09103 Lipid metabolism	ko00071 Fatty acid degradation	1.2338	0.0584	4	16	*hcaD*, ABUW_1624	ABUW_1150, ABUW_1151
K09104 Nucleotide metabolism	ko00230 Purine metabolism	1.4204	0.0380	1	51	-	*guaD1*
K09106 Metabolism of other amino acids	ko00470 D-Amino acid metabolism	0.3091	0.4908	2	17	ABUW_1519, ABUW_2787	-
K09108 Metabolism of cofactors and vitamins	ko00740 Riboflavin metabolism	0.6109	0.2449	2	10	*msuE*	ABUW_2498
K09110 Biosynthesis of other secondary metabolites	ko00996 Biosynthesis of various alkaloids	1.0218	0.0951	1	1	*hcaA*	-
**K09130 Environmental Information Processing**							
K09131 Membrane transport	ko02010 ABC transporters	0.4935	0.3210	6	49	ABUW_2820	ABUW_1570, ABUW_1569, *hisJ*, *hisM, hisQ*
	ko03070 Bacterial secretion system	0.8226	0.1505	5	30	-	ABUW_2618, ABUW_2578, *icmF,* ABUW_2566, *clpV*
K09132 Signal transduction	ko02020 Two-component system	0.3124	0.4871	5	60	ABUW_1507, *irlR*, *kdpB*	*pfeA, wza*
**K09140 Cellular Processes**							
K09145 Cellular community—prokaryotes	ko02025 Biofilm formation—*Pseudomonas aeruginosa*	3.5291	0.0003	9	25	-	ABUW_2572, *icmF*, ABUW_2567, ABUW_2568, ABUW_2580, ABUW_2579, ABUW_2578, ABUW_2575, *clpV*
	ko02026 Biofilm formation—*Escherichia coli*	0.4087	0.3902	2	14	-	*wza,* ABUW_1145
**K09150 Organismal Systems**							
K09152 Endocrine system	ko03320 PPAR signaling pathway	1.1288	0.0743	2	5	*desC*, *hcaD*	-
**K09160 Human Diseases**							
K09167 Endocrine and metabolic disease	ko04936 Alcoholic liver disease	2.4985	0.0032	3	4	*desC*, *hcaD*	ABUW_1150
K09171 Infectious disease: bacterial	ko05131 Shigellosis	1.0218	0.0951	1	1	-	ABUW_2052

## Data Availability

The data are contained within the article and supplementary materials. Sequence data reported have been deposited in the NCBI Sequence Read Archive (SRA) database under BioProject ID: PRJNA992059 with the accession numbers, SRR25167735–SRR25167740.

## Supplementary Materials

The following supporting information can be downloaded at: https://www.mdpi.com/article/10.3390/antibiotics12071185/s1, Table S1: Total number of raw reads, reads after trim, and reads mapped generated after RNA-sequencing. Table S2: Total of 3868 genes expressed mapped in clinical carbapenem-resistant *Acinetobacter baumannii* strains. Table S3: Total of 366 DEGs identified in pellicle-forming clinical carbapenem-resistant *Acinetobacter baumannii* strains. Table S4: Clusters of Orthologous Categories of identified DEGs in pellicle-forming carbapenem-resistant *Acinetobacter baumannii* strains. Table S5a: Total enriched Gene Ontology terms in DEGs identified in pellicle-forming carbapenem-resistant *Acinetobacter baumannii* strains. Table S5b: Top 27 GO enriched terms and their respective enrichment score, *p*-value, DEGs count, class and genes list. Table S6a: Total of 55 KO terms enriched from KEGG enrichment. Table S6b: Top 30 enriched KO terms.
